# Isocucurbitacin B inhibits glioma growth through PI3K/AKT pathways and increases glioma sensitivity to TMZ by inhibiting hsa-mir-1286a

**DOI:** 10.20517/cdr.2024.01

**Published:** 2024-05-08

**Authors:** Mingyu Han, Junsha An, Sui Li, Huali Fan, Li Wang, Qing Du, Junrong Du, Yuxin Yang, Yuqin Song, Fu Peng

**Affiliations:** ^1^Department of Pharmacology, Key Laboratory of Drug-Targeting and Drug Delivery System of the Education Ministry, Sichuan Engineering Laboratory for Plant-Sourced Drug and Sichuan Research Center for Drug Precision Industrial Technology, West China School of Pharmacy, Sichuan University, Chengdu 610041, Sichuan, China.; ^2^Department of Epidemiology and Health Statistics, West China School of Public Health and West China Fourth Hospital, Sichuan University, Chengdu 610041, Sichuan, China.; ^3^Integrated Traditional Chinese and Western Medicine Department, Sichuan Clinical Research Center for Cancer, Sichuan Cancer Hospital & Institute, Sichuan Cancer Center, Affiliated Cancer Hospital of University of Electronic Science and Technology of China, Chengdu 610041, Sichuan, China.; ^4^Waigaoqiao Free Trade Zone, WuXi Biologics, Shanghai 214122, China.; ^5^Livzon Pharmaceutical Group Inc, Zhuhai 519090, Guangdong, China.; ^6^Chongqing Western Biomedical Technology Co. Ltd., Chongqing 400039, China.

**Keywords:** Isocucurbitacin B, glioma, network pharmacology, hsa-mir-1286a, drug sensitivity

## Abstract

**Aim:** Glioma accounts for 81% of all cancers of the nervous system cancers and presents one of the most drug-resistant malignancies, resulting in a relatively high mortality rate. Despite extensive efforts, the complete treatment options for glioma remain elusive. The effect of isocucurbitacin B (isocuB), a natural compound extracted from melon pedicels, on glioma has not been investigated. This study aims to investigate the inhibitory effect of isocuB on glioma and elucidate its underlying mechanisms, with the objective of developing it as a potential therapeutic agent for glioma.

**Methods:** We used network pharmacology and bioinformatics analysis to predict potential targets and associated pathways of isocuB in glioma. Subsequently, the inhibitory effect of isocuB on glioma and its related mechanisms were assessed through Counting Kit-8 (CCK-8), wound healing, transwell, Western blot (WB), reverse transcription-quantitative polymerase chain reaction (RT-qPCR), and other in vitro experiments, alongside tumor formation experiments in nude mice.

**Results:** Based on this investigation, it suggested that isocuB might inhibit the growth of gliomas through the PI3K-AKT and MAPK pathways. Additionally, we proposed that isocuB may enhance glioma drug sensitivity to temozolomide (TMZ) via modulation of hsa-mir-1286a. The CCK-8 assay revealed that isocuB exhibited inhibitory effects on U251 and U87 proliferation and outperformed TMZ. Wound healing and transwell experiments showed that isocuB inhibited the invasion and migration of U251 cells by suppressing the activity of MMP-2/9, N-cadherin, and Vimentin. The TdT-mediated dUTP-biotin nick end labeling (TUNEL) and flow cytometry (FCM) assays revealed that isocuB induced cell apoptosis through inhibition of BCL-2. Subsequently, we conducted RT-qPCR and WB experiments, which revealed that PI3K/AKT and MAPK pathways might be involved in the mechanism of the inhibition isocuB on glioma. Additionally, isocuB promoted the sensitivity of glioma U251 to TMZ by inhibiting hsa-mir-1286a. Furthermore, we constructed TMZ-resistant U251 strains and demonstrated effective inhibition by isocuB against these resistant strains. Finally, we confirmed that isocuB can inhibit tumor growth *in vivo* through experiments on tumors in nude mice.

**Conclusion:** IsocuB may protect against glioma by acting on the PI3K/AKT and MAPK pathways and promote the sensitivity of glioma U251 to TMZ by inhibiting hsa-mir-1286a.

## INTRODUCTION

The most challenging malignancies worldwide encompass stomach, lung, liver, colorectal, esophageal, breast cancer, and glioma. Gliomas account for 81% of central nervous system (CNS) malignancies and pose significant therapeutic challenges. They are classified into four grades (1-4) ranging from low to high grade^[1,2]^. According to the fifth edition of the WHO classification of tumors of the central nervous system, adult-type diffuse gliomas, pediatric-type diffuse low-grade gliomas, pediatric-type diffuse high-grade gliomas, and circumscribed astrocytic gliomas were featured^[3]^. There are numerous therapeutic methods for gliomas, which are mainly categorized into surgery, immunotherapy, targeted therapy, electric field therapy, and chemophototherapy^[4,5]^. At present, the predominant approach globally involves maximal surgical resection supplemented with temozolomide (TMZ) and radiotherapy. However, this regimen generally yields a dismal^[6]^.

Modern pharmaceutical systems commonly use natural remedies to extract active ingredients with pharmacological properties. 60,000 years ago, plants were used as medicines, reflecting a wide range of pharmacological properties, including anticancer activity. Numerous natural compounds^[7]^, such as quercetin^[8]^, isoliquiritigenin^[9]^, and lycopene^[10]^, have apparent efficacy in inhibiting tumorigenesis, inhibiting tumor metastasis, and controlling tumor growth. Isocucurbitacin B (IsocuB) is isolated from the stalked tips of melons in the angiosperm family Cucurbitaceae. Natural cucurbitines exhibit various biological and pharmacological activities. Almost all the Cucurbitaceae families consist of approximately 229 species. Cucurbitacin is a class of highly oxidized tetracyclic triterpenoids with potent anticancer activity. Among its eight components of cucurbitacins with notable anticancer activity are cucurbitacin B, D, E, I, C, II, A, L-glucoside, S and R^[11]^. Its enantiomeric isomer, Cucurbitacin B^[12]^, has long been known for its role in lung cancer^[13,14]^, colorectal cancer^[15]^, colon cancer^[16]^, and glioma^[17]^. Melon pedicle was first documented in Shennong’s Herbal Classic, which describes its function in eliminating dampness and inducing vomiting. Furthermore, many ancient texts, such as “Shengji General Record”, “Qianjin Yaofang”, and “Ancient and Modern Medical Systems” also documented the use of melon pedicels for treating toothache, malaria, and hemorrhoids. Modern research has revealed that melon stalks have antitumor, hepatoprotective, and other beneficial effects. Clinical reports also indicate the utilization of melon stalks in the treatment of acute jaundice, infectious hepatitis, chronic rhinitis, chronic hepatitis, primary liver cancer, and other conditions. However, the anti-glioma effects of isocuB have not been proven.

While much is known about the molecular, structural, energetic, and chemical aspects of drug-target interactions, challenges remain in the selection and definition of targets, hindering the advancement of pharmacology and pharmacotherapy to an exact science^[18]^. The development of network pharmacology and bioinformatics will not only reduce the cost of drug development, but also shorten the time required for drug development. Furthermore, molecular docking techniques enable the prediction of the binding affinities and conformations of receptors and ligands^[19,20]^. This has been extremely helpful in our research. A deeper comprehension of the role of microRNAs (miRNAs) in development and disease, particularly in cancer, renders them attractive tools and targets for innovative therapeutic approaches. Functional studies have indicated the causal role of miRNA dysregulation in many cancers. MiRNAs show promise in preclinical development as tumor suppressors or oncogenes miRNA mimics, and molecules targeting miRNAs^[21]^.

The aim of our study was to predict the target genes and pathways of isocuB against glioma using network pharmacology and data analysis. Afterwards, we validated these predictions through Counting Kit-8 (CCK-8), wound healing assay, transwell invasion, TdT-mediated dUTP-biotin nick end labeling (TUNEL) staining, reverse transcription-quantitative polymerase chain reaction (RT-qPCR), WB, and tumor growth experiments in nude mice. Then, we further investigated and predicted the relationship between miRNA and resistance to the drug TMZ. Our study demonstrated that isocuB exerts its inhibitory effects on glioma by regulating specific pathways and gene expression, suggesting a novel and effective treatment approach for glioma.

## METHODS

### Network pharmacology and databases analysis

#### Predicting the target genes of isocuB

In this study, we used the Comparative Toxicogenomics database (http://ctdbase.org/), the PharmMapper database (http://www.lilab-ecust.cn/pharmmapper/), and the SwissTargetPrediction database (https://www.sib.swiss/). We set our filter to “number of interactions > 1” to identify isocuB targets. In total, 300 potential targets predicted for isocuB were identified.

#### Search related therapeutic target genes of glioma

We simultaneously used “glioma” as a keyword in the Online Catalog of Human Genes and Disorders (OMIM) database (https://www.omim.org/), the CTD database, and the GeneCards database (https://www.genecards.org). We set the filter to “Relevance score ≥ 10” to search for therapeutic targets associated with glioma. After collating the results, we identified 22,320 relevant therapeutic targets by removing duplicate values.

#### Obtaining intersection target genes of isocuB and glioma

We utilized the Bioinformatics & Evolutionary Genomics website (http://bioinformatics.psb.ugent.be/webtools/Venn/) to identify common targets at the intersection of the aforementioned glioma therapeutic targets and isocuB target genes. Subsequently, we generated a Venn diagram.

#### Protein-protein interaction network construction

We imported the previously obtained intersection target genes of isocuB and glioma into the Search Tool for the Retrieval of Interacting Genes (STRING) database (https://string-db.org/). We specified the species as “Homo sapiens” and selected the parameter with the highest confidence level of 0.900 to retrieve a related file in the “.tsv” format. We then conducted a topological analysis using the Cytoscape software (https://cytoscape.org/) to identify the core genes with the strongest correlation.

#### Analysis of biological functions and pathways

To predict the correlation pathway and function of isocuB in glioma, we used the Database for Annotation, Visualization, and Integrated Discovery (DAVID) (https://david.ncifcrf.gov/) to conduct Gene Ontology (GO) process and Kyoto Encyclopedia of Genes and Genomes (KEGG) pathway enrichment analyses (*P* < 0.05). Based on the above results, we utilized the R programming language to visualize the GO and KEGG pathway analyses. GO enrichment involves analyses of molecular function (MF), cell component (CC), and biological process (BP). KEGG is a bioinformatics resource used to identify a comprehensive list of genes that significantly impact metabolic pathways.

#### Molecular docking analysis

To improve the prediction of the relationship between isocuB and the top five related genes in the protein-protein interaction (PPI) analysis, we performed molecular association prediction. First, we obtained the structure of the gene from the Protein Data Bank (PDB) (http://www.rcsb.org) and acquired the 3D structure of isocuB from the PubChem database (https://pubchem.ncbi.nlm.nih.gov/). Next, we removed the hydrogen and ligands and dehydrated the hub gene proteins using the PyMOL 1.7.x software (http://www.pymol.org/). The AutoDock Tools 1.5.6 software (https://autodock.scripps.edu/) was used to convert the file to the PDBQT format. The PyMOL software was used to visualize the docking results.

#### Research based on database data

The hub genes (*RXRα*, *AKT1*, *ESR1*, *MAPK1*, and *HSP90AA1*) were identified, and their mRNA expression levels in various tumor tissues were analyzed using the University of Alabama at Birmingham (UALCAN) platform (https://ualcan.path.uab.edu/analysis.html). We analyzed the mRNA expression levels in glioma and related factors using the Chinese Glioma Genome Atlas (CGGA) database (http://www.cgga.org.cn/). The top five gene mutations and copy number variants were analyzed using the cBioPortal for Cancer Genomics database (https://www.cbioportal.org/). The CGGA database was utilized to analyze the correlation between the mRNA expression levels of each gene and the clinical parameters and prognosis associated with glioma. The GEPIA database, which includes all glioma data and group cutoffs from The Cancer Genome Atlas (TCGA), was selected as the reference. The mRNA expression levels of each gene in the gliomas were confirmed using GEPIA. The mRNA expression levels of each gene in gliomas were verified, and their relationship with prognosis was analyzed.

### Experimental verification

#### Cell culture and drug

Human glioma cells (U251 and U87, obtained from Shanghai Zhong Qiao Xin Zhou Biotechnology Co., Ltd., China) were cultured in complete DMEM (Shanghai Zhong Qiao Xin Zhou Biotechnology Co., Ltd., China). The environment for cultured cells needs to meet three conditions simultaneously: water saturation, a temperature of 37 °C, and 5% CO_2_ in the air. IsocuB, with a purity of ≥ 99.0%, was obtained from Chengdu Must Bio-technology Co., Ltd., China. TMZ was purchased from MCE in China with a purity of ≥ 99.0%. The drugs were dissolved in dimethyl sulfoxide (DMSO). The DMSO was diluted to less than 0.1% to ensure that it had no effect on the cells.

#### Reagents

CCK-8 (Dojindo, Japan), Matrigel Matrix (BD Biosciences, USA), paraformaldehyde (Solarbio, China), crystal violet (Sigma, USA), BeyoECL Plus (PIERCE, USA), TUNEL Assay Kit (Yesen, China), Annexin v-FITC/PI Apoptosis Kit (Boster, China), TRIzol Solution (Invitrogen, USA), Reverse Transcription System (Promega, USA), DEPC (Sigma, Germany), GoTaq® qPCR Master Mix (Promega, USA), Primers (Sangon Biotech, China), Bovine Serum Albumin (BSA) and BAC Protein Quantifier Kit (Beyotime Biotechnology, China), N-cadherin, Vimentin, BCl-2, Hsp90, p38-MAPK, p-MAPK, MMP-2, MMP-9, PDK1, anti-AKT, anti-p-AKT (CST, USA), p-STAT3 and STAT3 (Abclonal, China), β-actin (Servicebio, China), Bulge-Loop miRNA ORT-PCR Starter Kit, Human Bulge-Loop hsa-miR-1268a Primer Set, Bulge-Loop U6 gPCR Primer Set, Human microFF hsa-miR-1286 Inhibitor and microFF Inhibitor NC (RIBOBIO, China), micrON hsa-miR-1286 mimic (Genepharma, China) were used in this study.

#### Cell proliferation assay

We evaluated the effect of isocuB on glioma cell proliferation using a CCK-8 assay. We initially used 0.25% trypsin to digest U251 and U87 cells at the logarithmic growth stage and subsequently diluted them to a seeding density of 7 × 10^4^ cells/mL in 96-well plates. IsocuB was then diluted to seven concentrations (0.001, 0.01, 0.1, 0.5, 1, 5, and 25 µmol/L). TMZ was then diluted to seven concentrations (10, 50, 100, 200, 500, 1,000, and 2,000 µmol/L). These were added 12 and 24 h later. Finally, the CCK-8 reagent was added at 12 and 24 h for absorbance detection at 450 nm using a microplate reader. Triplicate experiments were performed independently {Cell viability = [(OD_Experiment group_ - OD_Blank_)/(OD_Control group_ - OD_Blank_)] × 100%}.

#### Wound healing assay

We assessed the impact of isocuB on glioma cell migration by conducting a wound-healing assay. We diluted the cell density of U251 to 2.5 × 10^5^ cells/mL and plated it into a 6-well plate. Five parallel lines (2 mL per well) were drawn beside each well with a marker. Then, 24 h later, the lines were drawn with a 200 µL pipette tip tilted at a 45° angle, washed three times with PBS, and DMEM dilution containing 1% FBS in three concentrations of isocuB (0, 0.01, and 0.1 µmol/L) was added. Finally, five visual fields were randomly selected at 12, 24, and 36 h to capture images and observe the migration of U251 cells. Triplicate experiments were performed independently.

#### Transwell invasion

To assess the invasive impact of isocuB on glioma cells, a transwell invasion assay was conducted. The diluted matrix adhesive was added to the upper chamber and placed in an incubator for 8 h to allow the matrix to form and absorb excess fluid. After 12 h of cell starvation, 5 × 10^4^ cells were seeded into the upper chamber with 100 μL of 0.2% BSA DMEM containing isocuB (0, 0.01, and 0.1 µmol/L), and 500 μL of complete DMEM medium was added to the lower chamber. After 24 h, non-migrated cells on the filter side of the upper chamber were removed using a cotton swab, fixed with 4% paraformaldehyde for 1 h, rinsed three times with PBS, and stained with 1 mL of crystal violet for 1 h. The transwell membrane was then covered with a cover glass, and the migrated cells were counted under a microscope. Triplicate experiments were performed independently.

#### TUNEL staining

The U251 cells in the growth stage were seeded in 6-well plates at a density of 2.5 × 10^5^ cells/mL, with 2 mL per well. The drug (0, 0.1, 0.5, and 1 µmol/L) was added after cell adhesion. After a 24-hour incubation, the cells were harvested using ethylenediaminetetraacetic acid-free pancreatic enzymes and washed twice with PBS. Each sample was supplemented with 50 µL of FITC-12-dUTP and incubated at 37 °C for 1 h, followed by testing using a machine. Triplicate experiments were performed independently.

#### Flow cytometry analysis of cell apoptosis

The U251 cells in the growth stage were seeded in 6-well plates at a density of 2.5 × 10^5^ cells/mL, with 2 mL per well. The drug (0, 0.1, 0.5, and 1 µmol/L) was added after cell adhesion. After a 24-hour incubation, the cells were harvested using ethylenediaminetetraacetic acid-free pancreatic enzymes and washed twice with PBS. 500 µL of 1× Annexin V Binding Buffer was added to each sample. Subsequently, 5 µL of annexin V-FITC staining solution and 5 µL of PI staining solution were added to each sample, incubated at room temperature for 5-15 min, and then analyzed by machine. Triplicate experiments were performed independently.

#### RT-qPCR analysis

To investigate the mechanism of action of isocuB in glioma, we used RT-qPCR. First, RNA was extracted from U251 cells treated with isocuB (0, 0.1, 0.5, 1 µmol/L) using TRIzol. RNA concentration and purity were determined using a miRNA protein quantification assay. The assay was performed according to the instructions provided with the SYBR® Green Real-time PCR Master Mix kit. Cycle threshold (Ct) was determined using quantitative PCR. The primer sequences for MMP-2, MMP-9, PDK1, RXRα, PPARα, Bcl-2, and β-actin are provided in Table 1. Additionally, we analyzed the RNA content of hsa-mir-1286a using U6 as the internal reference. Hsa-miR-1286a inhibitor and microRNA inhibitor NC were then added to the 6-well plate at a concentration of 100 µmol/L. RNA was extracted 24 h later, and the level of hsa-miR-1286a was detected. Moreover, hsa-miR-1286a mimic was added to the 6-well plate at a concentration of 100 µmol/L. RNA was extracted 24 h later, and the level of hsa-miR-1286a was detected. Triplicate experiments were performed independently.

**Table 1:** Primers used for RT-qPCR analysis

**Gene**	**Primer sequence**	
	**Forward (5’-3’)**	**Reverse (5’-3’)**
MMP-2	GATACCCCTTTGACGGTAAGA	CCTTCTCCCAAGGTCCATAGC
MMP-9	GGGACGCAGACATCGTCATC	TCGTCATCGTCGAAATGGGC
PDK1	CGTACGGCAATGGCTTTATC	AATCCCCTCCTGCAACTTCT
RXRα	ATGGACACCAAACATTTCCTG	GGGAGCTGATGACCGAGAAAG
PPARα	TTCGCAATCCATCGGCGAG	CCACAGGATAAGTCACCGAGG
Bcl-2	GGTGGGGTCATGTGTGTGG	CGGTTCAGGTACTCAGTCATCC
β-actin	AAAGCGGCTGTTAGTCACTGG	CGAGTCATTGCATACTGTCCAT

RT-qPCR: Reverse transcription-quantitative polymerase chain reaction; MMP2/9: matrix metalloproteinase 2/9; PDK1: 3-phosphoinositide-dependent protein kinase 1.

#### WB analysis

To further investigate the mechanism of action of isocuB on glioma, the proteins in U251 cells treated with different concentrations of isocuB (0, 0.1, 0.5, 1 µmol/L) for 24 h were extracted using RIPA lysis. The extracted protein solution was then assayed using the BCA kit and denatured using SDS. Proteins were separated at a constant voltage of 110 V for 90 min and then transferred to a PVDF membrane (Mannheim, Germany). After blocking with 5% skim milk powder, the primary antibodies for MMP-2, MMP-9, p-PI3K, PI3K, p-AKT(S473), AKT, p-STAT3, STAT3, RXRα, PDK1, p-MAPK1/3, MAPK1/3, N-cadherin, Vimentin and Bcl-2 (1:1000) were added and incubated overnight at 4 °C. After washing with TBST, appropriate secondary antibodies were added, incubated at 37 °C for 1 h, and then washed with TBST and TBS. Luminescent agents were then added to facilitate detection in the chemiluminescence imaging system, and further analysis was performed using ImageJ software. Triplicate experiments were performed independently.

#### Establishment of U251/TMZ resistant strains

Cells in the logarithmic growth phase (80%-90%) were treated with drugs at a low initial concentration (1/10-1/5 of IC_50_ of the parent cell line is recommended), and cultured in an incubator at 37 °C and 5% CO_2_. When the cell density reached 50%, the culture medium was abandoned, PBS was cleaned twice, and the drug-free medium was replaced for further culture. When the cell growth density recovered to 80%-90%, the above drug treatment was repeated 6-8 times. The final drug concentration was stable and the drug-resistant cell line was obtained. The IC_50_ values of drug-resistant cell lines were determined, and the resistance index (RI) was calculated using the formula: RI = IC_50_ of drug-resistant cell lines/IC_50_ of parental cell lines. The RI grades range from 1 to 5, indicating low drug resistance, 5 to 15, indicating moderate resistance, and above 15, indicating high resistance. Triplicate experiments were performed independently.

#### Inhibitory effect of isocuB on U251/TMZ resistant strains

The primary physiological characteristics of tumor cells include proliferation, invasion, and migration. We evaluated the effect of isocuB on glioma cell proliferation using the CCK-8 assay. We initially used 0.25% trypsin to digest U251/TMZ-resistant strain cells at the logarithmic growth stage and subsequently diluted them to a seeding density of 7 × 10^4^ cells/mL in 96-well plates. TMZ was then diluted to seven concentrations (10, 50, 100, 200, 500, 1,000, and 2,000 µmol/L). IsocuB was then diluted to seven concentrations (0.001, 0.01, 0.1, 0.5, 1, 5, and 25 µmol/L). These were added 24 h later. Finally, the CCK-8 reagent was added at 24 h for absorbance detection at 450 nm using a microplate reader. Triplicate experiments were performed independently {Cell viability = [(OD_Experiment group_ - OD_Blank_)/(OD_Control group_ - OD_Blank_)] × 100%}.

#### Transfection of hsa-mir-1286a inhibitor increased the sensitivity of glioma U251 to TMZ

We first used 0.25% trypsin to digest U251 cells at the logarithmic growth stage, and then diluted them to a seeding density of 7 × 10^4^ cells/mL in 96-well plates. Human microRNA hsa-mir-1286a inhibitor was then added to the 6-well plate at a concentration of 100 µmol/L, and RNA was extracted 24 h later. TMZ was then diluted to seven concentrations (10, 50, 100, 200, 500, 1,000, and 2,000 µmol/L) and added 24 h later. Finally, the CCK-8 reagent was added after 24 h for absorbance measurement at 450 nm using a microplate reader. Triplicate experiments were performed independently {Cell viability = [(OD_Experiment group_ - OD_Blank_)/(OD_Control group_ - OD_Blank_)] × 100%}.

#### Transfection of hsa-miR-1286a mimic inhibited the sensitivity of glioma U251 to TMZ

We initially used 0.25% trypsin to digest U251 cells at the logarithmic growth stage, and subsequently diluted them to a seeding density of 7 × 10^4^ cells/mL in 96-well plates. A hsa-miR-1286a mimic was then added to the 6-well plate at a concentration of 100 µmol/L, and RNA was extracted 24 h later. TMZ was then diluted to seven concentrations (10, 50, 100, 200, 500, 1,000, and 2,000 µmol/L) and added 24 h later. Finally, the CCK-8 reagent was added after 24 h for absorbance measurement at 450 nm using a microplate reader. Triplicate experiments were performed independently {Cell viability = [(OD_Experiment group_ - OD_Blank_)/(OD_Control group_ - OD_Blank_)] × 100%}.

#### Tumor formation experiment in nude mice

In our study, 16 six-week-old specific pathogen-free (SPF) BALB/c nude mice were initially transplanted with 5 × 10^6^ U251 cells in a 100 µL PBS volume in the right upper axilla. The mice were then divided into two groups (IsocuB group at 2 mg/kg and DMSO group) on day 6 after transplantation. During the experiment, tumor size was measured daily in both groups of nude mice. On day 18, the masses were removed and weighed. We assessed and compared the supervisors. These studies were conducted in compliance with the ARRIVE guidelines^[22,23]^ and the National Research Council Guide for the Care and Use of Laboratory Animals. All animal experiments followed the regulations of ethics committee of the Experimental Animal Administration of Sichuan University (NO. K2024006).

#### Statistical analysis

To ensure the accuracy of the experimental results, each set of experiments was repeated at least three times. The final data were presented as the mean ± standard deviation (SD), and the differences were statistically analyzed using GraphPad software (*P* < 0.05 was considered statistically significant).

## RESULTS

### Network pharmacology and databases analysis

**Figure 1:**
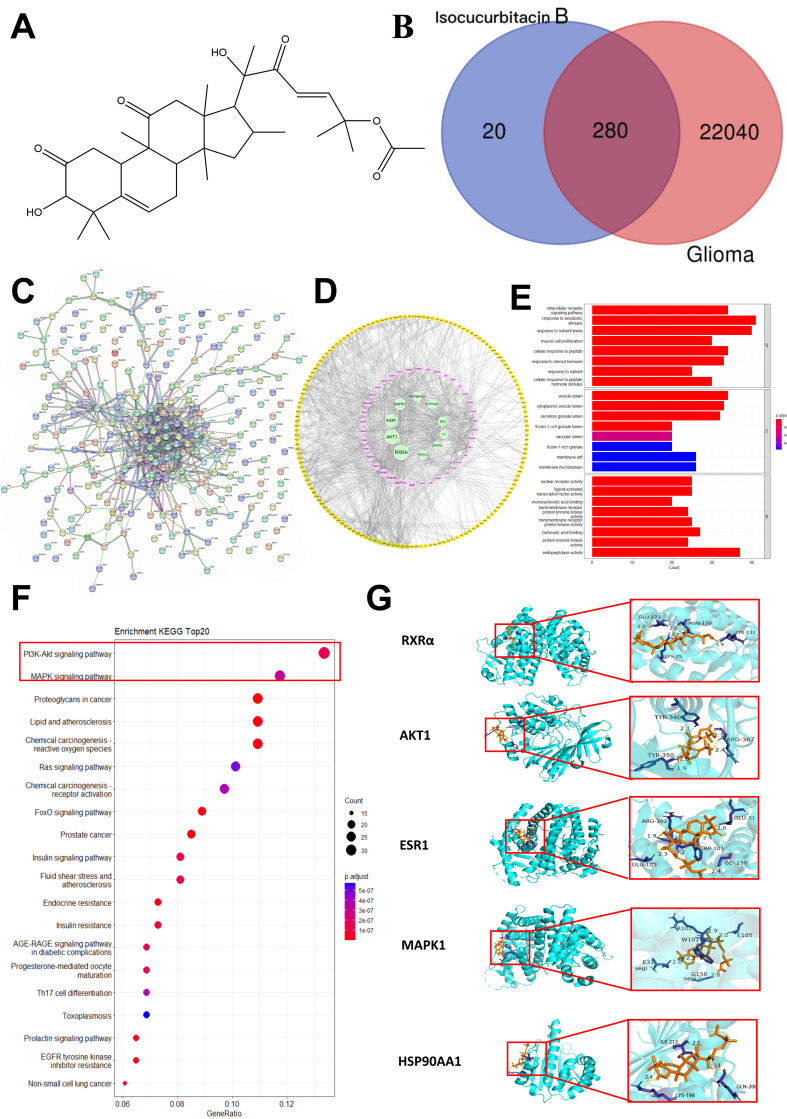
(A) IsocuB, drawn using Chemdraw; (B) Obtain 280 intersection target genes for isocuB and glioma; (C) STRING database of the 280 potential therapeutic targets; (D) PPI network of the 280 potential therapeutic targets; (E) The top 10 significant enrichment terms in BP, CC, and MF are shown in a GO enrichment bubble map containing 280 intersecting targets; (F) The top 20 most significantly enriched pathways Bar diagram of KEGG pathway enrichment analysis with 280 cross-target sites; (G) The result diagram of molecular docking. The yellow 3D structural formula represents isocuB; the blue bond represents the hydrogen bond at the binding site. IsocuB: Isocucurbitacin B; STRING: search tool for the retrieval of interacting genes; PPI: protein-protein interaction; BP: biological process; CC: cell component; MF: molecular function; GO: Gene Ontology; KEGG: Kyoto encyclopedia of genes and genomes.

In this study, the targets of isocuB [Figure 1A] were predicted using the CTD, Swiss Target Prediction, and PharmMapper databases. A total of 301 predicted targets were identified as potential targets of isocuB. In this study, we identified potential therapeutic target genes for glioma using the GeneCards, OMIM, and CTD databases. The results were compiled, yielding 22,320 relevant therapeutic targets. We imported the 280 common genes [Figure 1B, Supplementary Table 1] into the STRING database and obtained the PPI information for 280 nodes and 631 edges [Figure 1C]. The corresponding PPI information was imported into Cytoscape software. The analysis revealed that the top five genes affected by isocuB in glioma were RXRα, AKT1, ESR1, MAPK1, and HSP90AA1 [Figure 1D, Supplementary Table 2]. A total of 826 GO analyses were performed, and the first 10 enrichment results are visualized in Figure 1E based on the *P* value. The analyses were mainly divided into three categories: (1) 278 BP, including response to protein binding, peptidyl-tyrosine phosphorylation, and protein autophosphorylation; (2) 279 CC, including cytosol, extracellular exosome, and extracellular region; (3) 279 MF, including signaling receptor activator activity and identical protein binding activity. KEGG analysis revealed 152 major pathways, with the 20 most significantly enriched pathways shown in Figure 1F. These pathways included the PI3K/AKT signaling pathway, MAPK signaling pathway, and proteoglycans in cancer signaling pathways. The top five targets were found to be highly connected in the network according to the PPI analysis, located at the core of the network, and identified as the most important nodes. The binding affinities of isocuB to RXRα (3HOA), AKT1 (4EJN), ESR1 (6V8T), MAPK1 (6G54), and HSP90AA1 (4BQG) were -7.41, -6.67, -7.65, -7.95, and -7.68 kcal/mol [Figure 1G]. In addition, a coupling fraction of less than 0 kcal/mol indicates that the component can spontaneously bind to the target, less than -4.25 kcal/mol indicates a good affinity coupling, and less than -7 kcal/mol is considered as strong affinity coupling^[24]^. The results indicated a strong binding activity between the main components and the hub gene (affinity < -6.00 kcal/mol).

**Figure 2:**
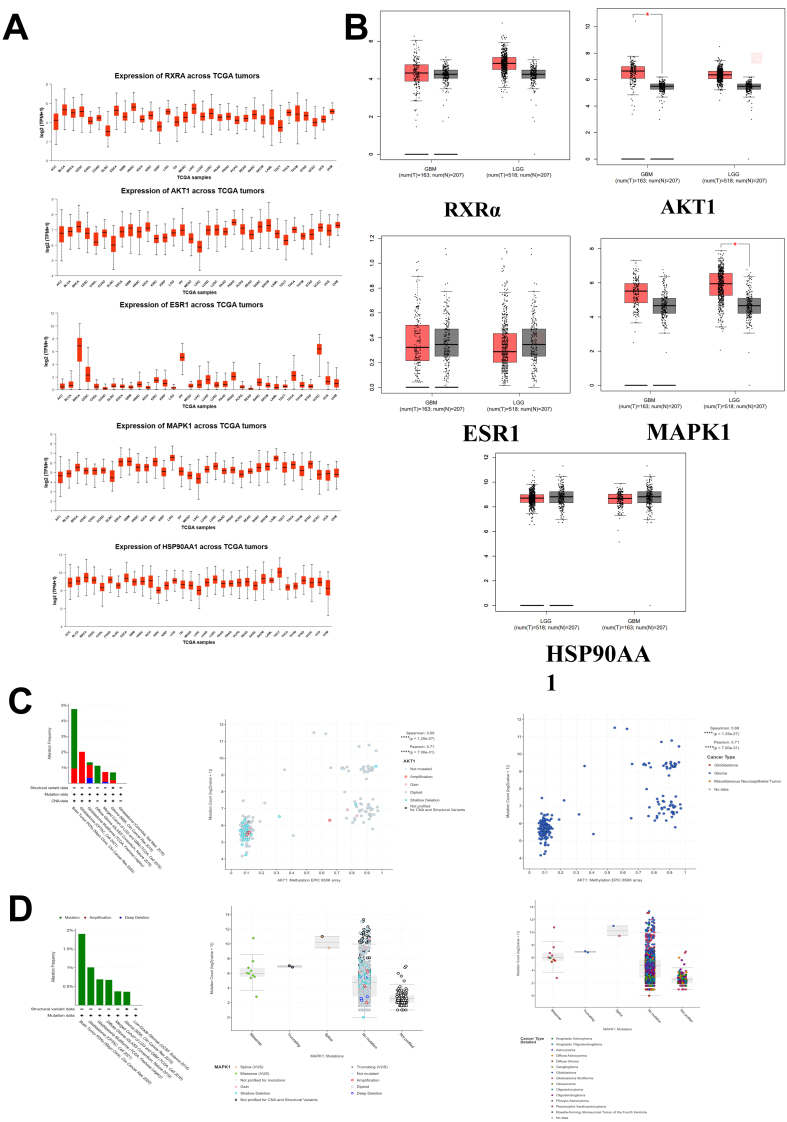
(A) mRNA expression levels in common tumor tissues; (B) The mRNA expression levels in common tumor tissues (^*^P < 0.05 *vs.* control group); (C) Mutation, deep deletion, and amplification of AKT1 in gliomas. Green, blue, and red indicate mutations, deep deletions, and amplifications, respectively. AKT1 methylation is negatively correlated with the expression of AKT1 in glioma tissues (^****^P < 0.0001 *vs.* control group). The AKT1 gene has a low methylation level in gliomas. (^****^P < 0.0001 *vs.* control group); (D) Mutations, deep deletions, and MAPK1 amplification in glioma cells. Green indicates mutations. Different types of MAPK1 mutations. Different types of MAPK1 mutation in glioma at different levels. AKT1: AKT serine/threonine kinase 1; MAPK1: mitogen-activated protein kinase 1.

Based on RNA-Seq data from the UALCAN database, a total of 7932 case samples were included. The mRNA expression of RXRα, AKT1, ESR1, MAPK1, and HSP90AA1 was analyzed in different tumor tissues. The mRNA expression levels of AKT1 and MAPK1 were relatively high in glioma, while those of RXRα, ESR1, and HSP90AA1 were relatively low [Figure 2A]. The mRNA expression of RXRα, AKT1, ESR1, MAPK1, and HSP90AA1 in tumor and normal tissues was analyzed using the GEPIA database. The mRNA expression of RXRα, ESR1, and HSP90AA1 did not show significant differences between tumor tissues and normal tissues [Figure 2B]. AKT1 and MAPK1 mRNA expression levels were significantly different in tumor and normal tissues in gliomas (*P <* 0.05) [Figure 2B]. According to the KEGG analysis, the top two pathways were PI3K-AKT and MAPK. Molecular docking showed a strong binding effect. The mRNA expression levels of AKT1 and MAPK1 were significantly different between normal and glioma tissues. Next, we analyzed the mRNA expression levels of MAPK1 and AKT1. Gene mutations and copy number variations of MAPK1 and AKT1 in gliomas were analyzed using the cBioPortal for Cancer Genomics database. Based on an analysis of the cBioPortal for Cancer Genomics database, seven studies included 3436 cases of glioma.

AKT1 contains mutations, deep deletions, and amplifications in gliomas. AKT1 exhibited the highest mutation rate of 3.81%, amplification rate of 2.02%, and deep deletion rate of 0.34% [Figure 2C]. A total of 189 samples from two studies were included in the methylation analysis. The results showed a negative correlation between AKT1 methylation and AKT1 gene expression in glioma tissues (*P <* 0.001), indicating that AKT1 gene expression may be epigenetically regulated. Copy number amplification of AKT1 was detected in three cases, while mild AKT1 copy number loss was detected in 29 cases. According to the pathological type, all 189 cases of GBM (100%) had low AKT1 methylation. Mutations in MAPK1 were identified in gliomas. MAPK1 exhibited the highest mutation rate (1.9%) without any apparent significant deletions or amplifications [Figure 2D]. Different mutations and different types of gliomas associated with MAPK1 are shown in Figure 2D.

**Figure 3:**
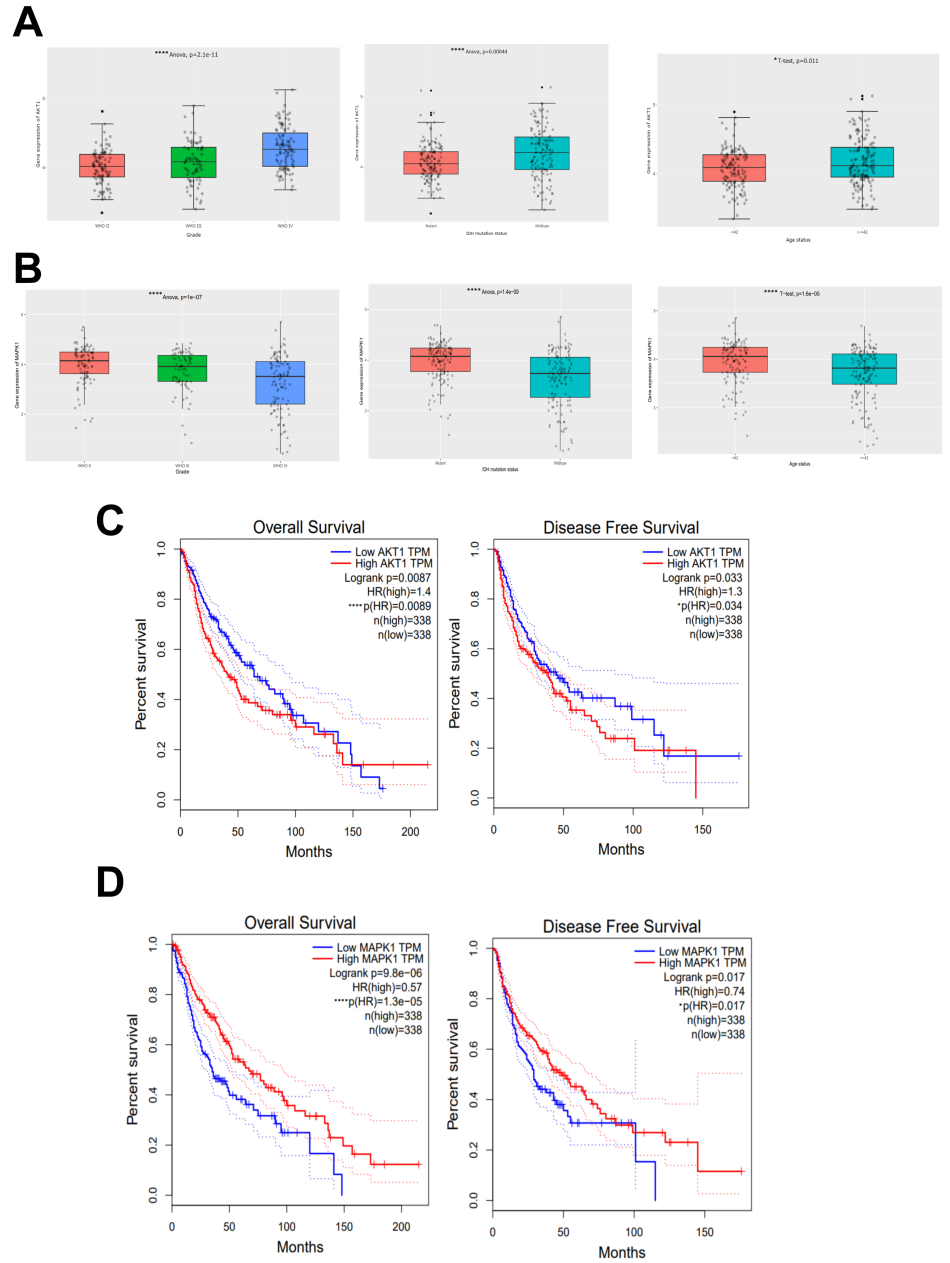
(A) Relationship between mRNA expression levels of AKT1 and clinicopathological features in patients with glioma. Significant differences in the expression of (WHO II, WHO III, and WHO IV) (^****^P < 0.0001 *vs.* control group). Significant differences among different states of IDH (wild type, mutant type) (^****^P < 0.0001 *vs.* control group). Significant differences among different age status (</≥ 42) (^*^P < 0.05 *vs.* control group); (B) Relationship between mRNA expression levels of MAPK1 and clinicopathological features in patients with glioma. Significant differences in the expression of (WHO II, WHO III, and WHO IV) (^****^P < 0.0001 *vs.* control group). Significant differences among different states of IDH (wild type, mutant type) (^****^P < 0.0001 *vs.* control group); Significant differences among different age status (</≥ 42) (^****^P < 0.0001 *vs.* control group); (C and D) The prognosis of the low AKT1 and MAPK1 expression groups was better than that of the high expression group (^*^P < 0.05, ^****^P < 0.0001 *vs.* control group). The disease-free survival of the low AKT1 and MAPK1 expression groups was better thanthat of the high expression group (^*^P < 0.05 *vs.* control group). AKT1: AKT serine/threonine kinase 1; IDH: isocitrate dehydrogenase; MAPK1: mitogen-activated protein kinase 1.

To investigate the relationship between AKT1 and MAPK1 mRNA expression and clinicopathologic parameters, we used the CGGA database, which comprises 325 cases from the glioma mRNA microarray database, for correlation analysis. The cutoff values for AKT1 and MAPK1 mRNA expression were 70.48 and 44.84, respectively. Values higher than the cutoff indicated high expression, and vice versa. The results indicated that AKT1 and MAPK1 were present in different tumor stages. There were statistically significant differences in the expression of glioma grades (WHO II, WHO III, and WHO IV) (*P <* 0.001) [Figure 3A and B], as well as statistically significant differences among different states of isocitrate dehydrogenase (IDH) (wild type, mutant type) (*P <* 0.001). There was a statistically significant difference among the various age groups (*P <* 0.05) [Figure 3A and B]. Expression of AKT1 and MAPK1 was correlated with WHO classification, IDH status, and age. The TCGA glioma data were retrieved from the GEPIA database. The group cutoff was selected as the median to divide the high expression group and the low expression group (cutoff-high = 50%; cutoff-low = 50%) for analyzing the mRNA expression of AKT1 and MAPK1 in glioma to verify their prognostic relationship. To validate the analysis results from the CGGA database and assess the prognostic significance of AKT1 and MAPK1 in glioma, survival analyses were performed using the GEPIA database. The results, using the Kaplan-Meier method, showed that there were 676 cases of TCGA glioma. The prognosis of the low AKT1 and MAPK1 expression groups was better than that of the high expression group, and the differences in overall survival (OS) between the two groups were statistically significant (*P* < 0.01, *P* < 0.0001). Disease-free survival of the low AKT1 and MAPK1 expression groups was better than that of the high expression group. The differences were significant (*P* < 0.05) [Figure 3C and D].

### Experimental verification results

#### IsocuB inhibits proliferation, migration and invasion and increases apoptosis

**Figure 4:**
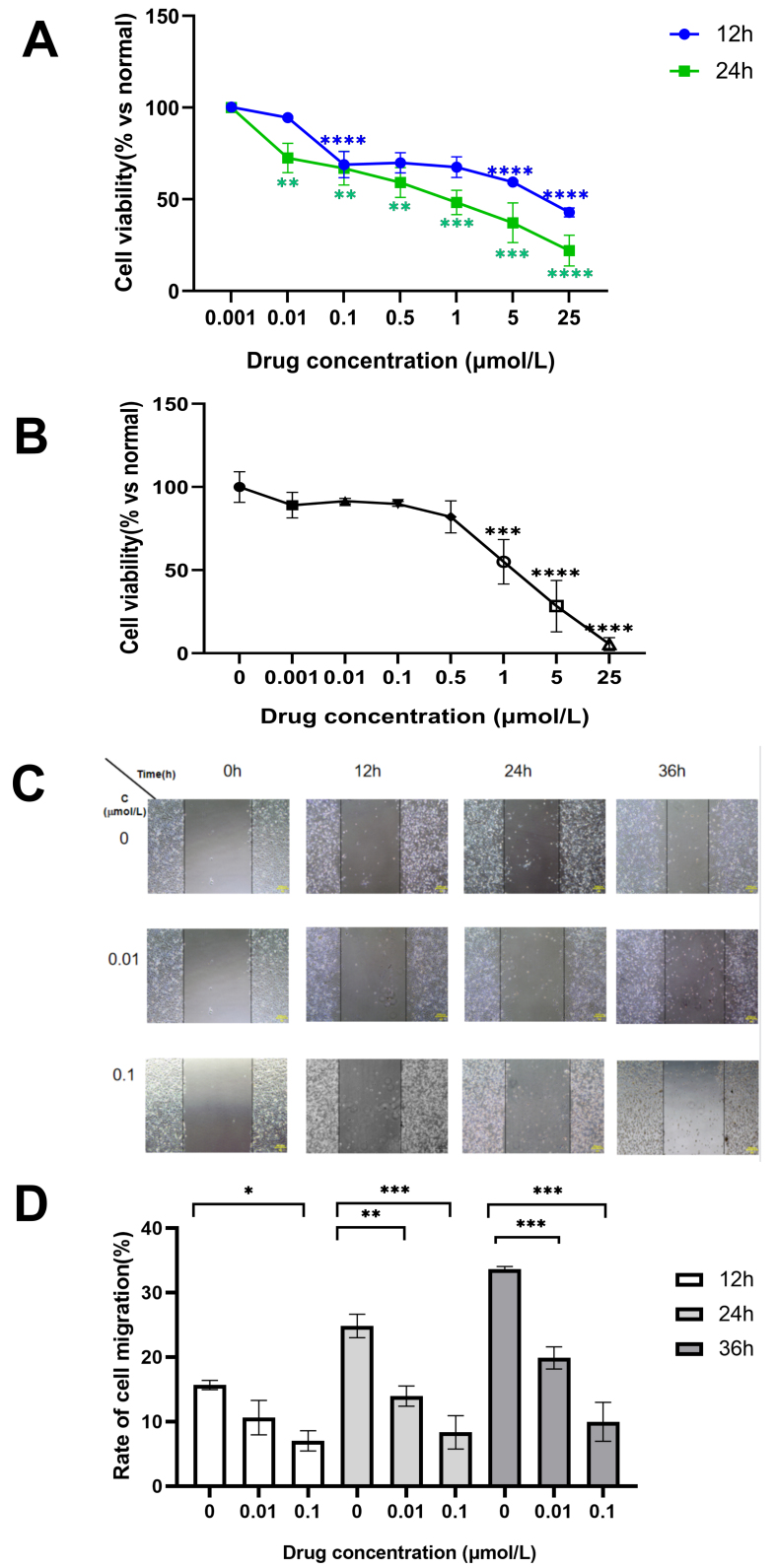
(A) The viability of U251 cells after 12 and 24 h of isocuB treatment (^**^P < 0.01, ^***^P < 0.001; ^****^P < 0.0001 *vs.* control group); (B) The viability of U87 cells after 24 h of isocuB treatment (^***^P < 0.001; ^****^P < 0.0001 *vs.* control group); (C and D) Cell mobility of U251 cells after12, 24 and 36 h of isocuB treatment (^*^P < 0.05, ^**^P < 0.01, ^***^P < 0.001 *vs.* control group). isocuB: Isocucurbitacin B.

Our experimental results demonstrated that isocuB had a concentration- and time-dependent effect on glioma cell proliferation in the CCK8 assay [Figure 4A]. The IC_50_ of isocuB-inhibited U251 cells is 0.79 µmol/L at 24 h and 10.54 µmol/L at 12 h. The IC_50_ of isocuB-inhibited U87 cells is 2.12 µmol/L at 24 h [Figure 4B]. Thus, the inhibitory effect of isocuB on U251 was much greater than that on U87; therefore, U251 was primarily used in our subsequent experiments. Our results demonstrate that isocuB inhibited glioma migration in a time- and dose-dependent manner, as illustrated in the wound healing assay [Figure 4C and D].

**Figure 5:**
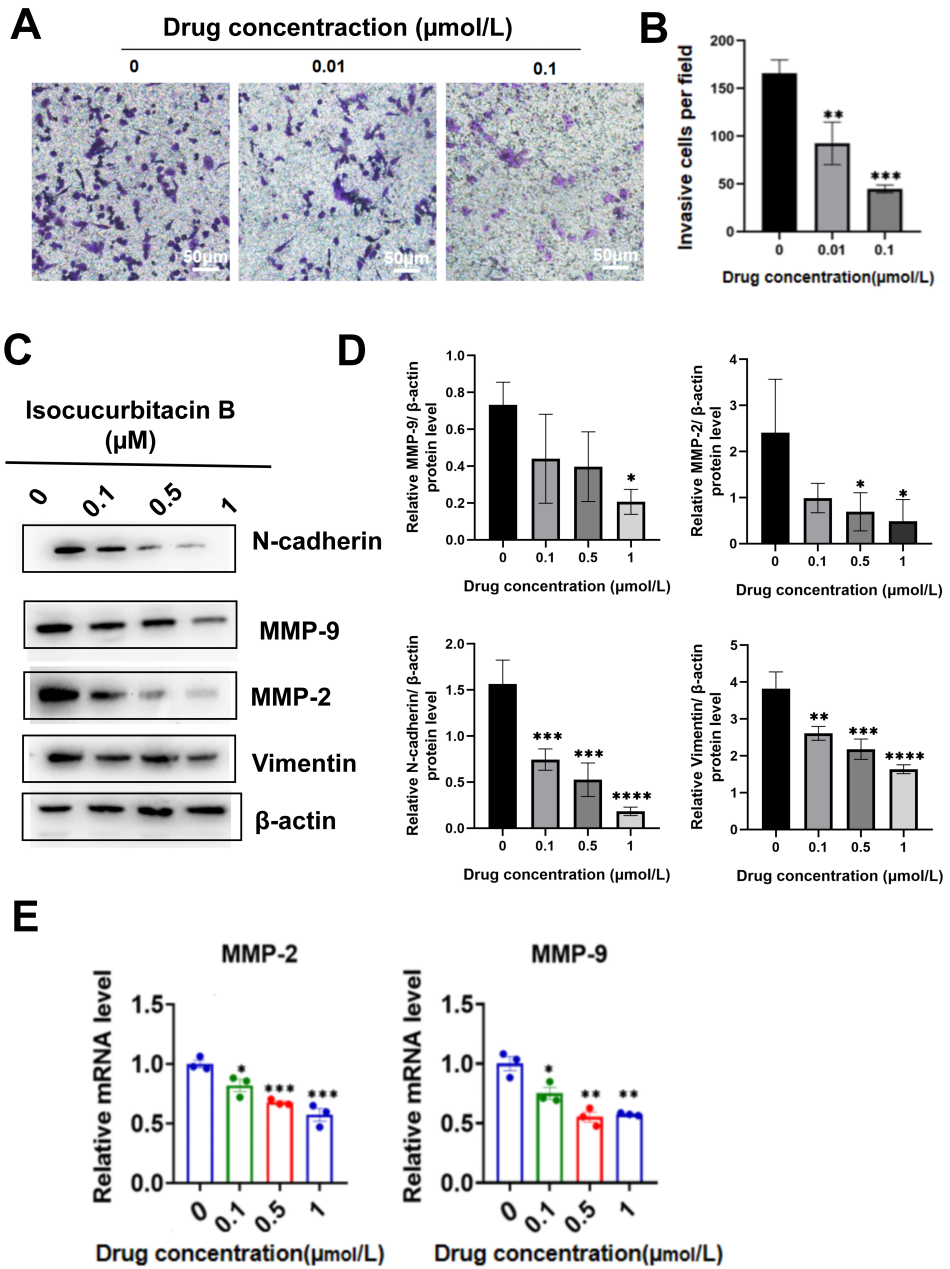
(A and B) The transwell invasion process is measured by the number of cells after 24 h of treatment (^**^P < 0.01, ^***^P < 0.001 *vs.* control group); (C and D) Protein expressions of MMP-2/9, N-cadherin, and Vimentin were analyzed using WB. Data are represented as mean ± SD (^*^P < 0.05, ^**^P < 0.01, ^***^P < 0.001, ^****^P < 0.0001 *vs.* control group *vs.* control group); (E) Expression of MMP-2 and MMP-9 RNA was detected using RT-qPCR (^*^P < 0.05, ^**^P < 0.01, ^***^P < 0.001 *vs.* control group). MMP-2/9: Matrix metalloproteinase 2/9; WB: Western blot; RT-qPCR: reverse transcription-quantitative polymerase chain reaction.

In addition, isocuB inhibited the invasion of glioma cells in the transwell invasion assay [Figure 5A and B]. The MMP family is associated with cancer cell survival, proliferation, apoptosis, invasion, and metastasis^[25]^. The mRNA and protein expression levels were significantly decreased after interference with isocuB in U251 cells [Figure 5C-E]. Epithelial-mesenchymal transition (EMT) is a process in which cells acquire invasive mesenchymal competence through a series of events that include loss of cellular connections, cytoskeletal reorganization, and remodeling of the extracellular matrix^[26]^. The results of the WB experiment results showed a significant decrease in the protein levels of N-cadherin and Vimentin. This indicated that isocuB could inhibit EMT and consequently inhibit glioma invasion [Figure 5C and D].

**Figure 6:**
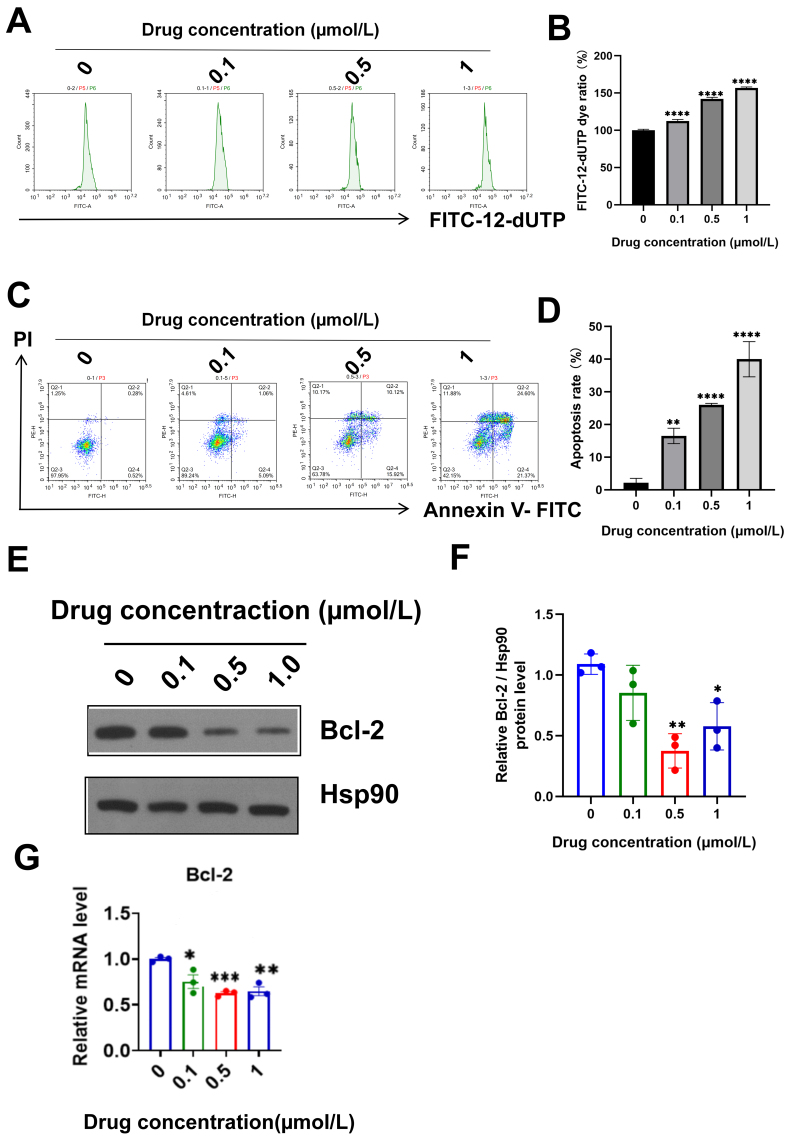
(A and B) Dye content of FITC-12-dUTP was detected by FCM (^****^P < 0.0001 *vs.* control group); (C and D) The apoptosis rate was increased by FCM. Q3-1 represents normal cells, Q3-4 represents viable apoptotic cells, Q3-2 represents late apoptotic cells, and Q3-1 represents mechanical destruction of cells (^**^P < 0.01, ^***^P < 0.001, ^****^P < 0.0001 *vs.* control group); (E and F) Protein expression of BCL-2 was analyzed using WB. Data are represented as mean ± SD (^*^P < 0.05, ^**^P < 0.01 *vs.* control group); (G) Expression of BCL-2 RNA was detected using RT-qPCR (*n* = 3) (^*^P < 0.05, ^**^P < 0.01, ^***^P < 0.001 *vs.* control group). FCM: Flow cytometry; BCL-2: B-cell lymphoma-2; WB: western blot; RT-qPCR: reverse transcription-quantitative polymerase chain reaction.

The TUNEL staining assay results indicated that isocuB promoted the apoptosis of U251 cells [Figure 6A and B]. Annexin V was used to detect phosphatidylserine (PS) exposure on the outer membrane of early apoptotic cells. However, annexin V cannot distinguish between poor cell death (middle and late apoptotic cells) and early apoptotic cells. PI can enter the necrotic cells (middle and late apoptotic cells) but is excluded from early apoptotic cells. This finding indicated that isocuB could induce the apoptosis of glioma U251 cells. The proportion of late apoptosis also increased with increasing drug concentration [Figure 6C and D]. WB analysis showed that the proportion of BCL-2 increased in U251 cells after administration [Figure 6E-G].

#### IsocuB inhibited the activation of the PI3K/AKT, MAPK, and STAT3 signaling pathways

**Figure 7:**
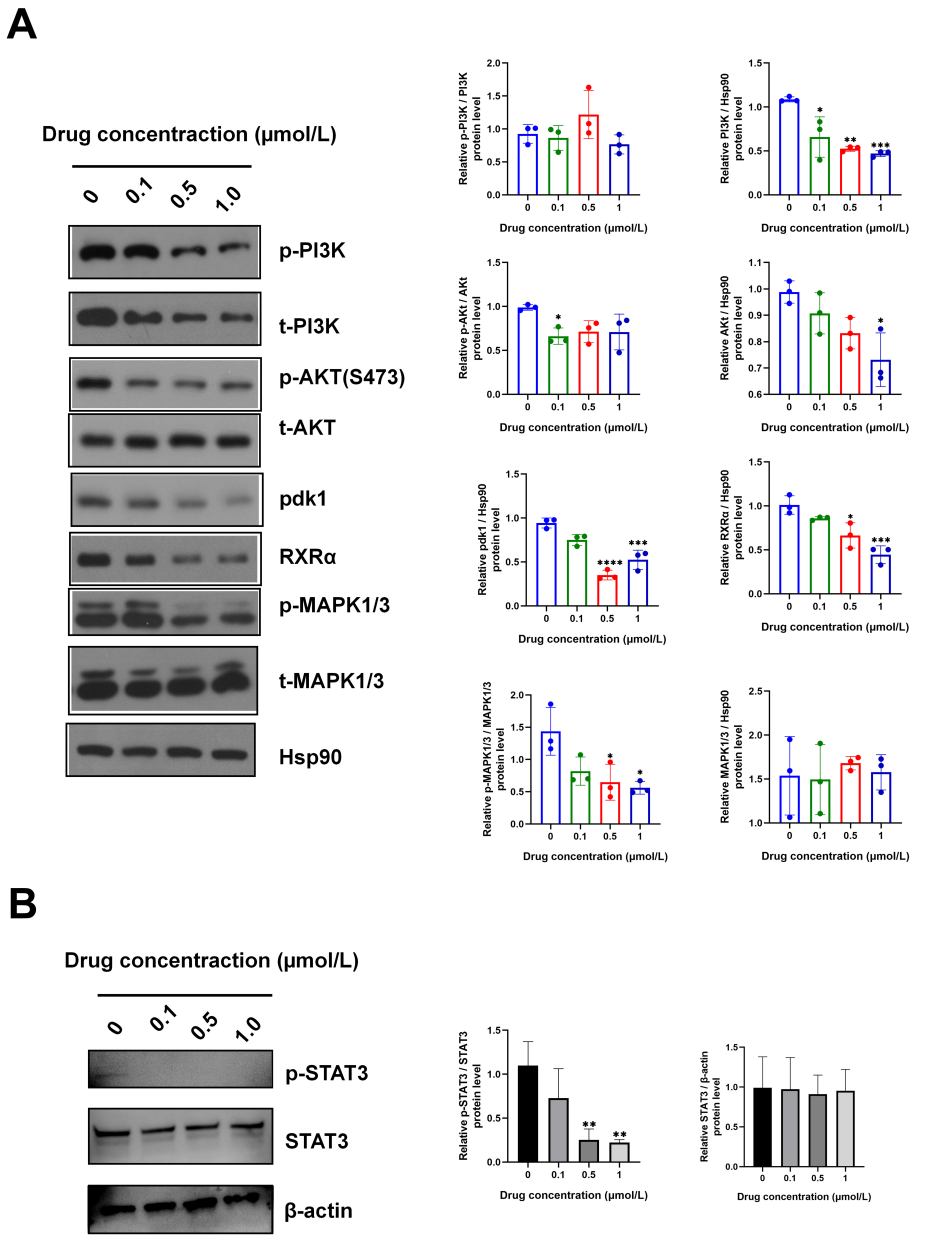
(A) Protein expressions of p-PI3K, PI3K, p-AKT(S473), t-AKT, RXRα, PDK1, p-MAPK1/3, MAPK1/3 and Bcl-2 were analyzed using WB. Data are represented as mean ± SD (^*^P < 0.05, ^**^P < 0.01, ^***^P < 0.001, ^****^P < 0.0001 *vs.* control group); (B) Protein expressions of p-STAT3, STAT3 were analyzed using WB. Data are represented as mean ± SD (^**^P < 0.01 *vs.* control group). PI3K: Phosphoinositide 3-kinase; AKT: AKT serine/threonine kinase; PDK1: 3-phosphoinositide-dependent protein kinase 1; MAPK: mitogen-activated protein kinase; WB: western blot; STAT3: signal transducer and activator of transcription 3.

**Figure 8:**
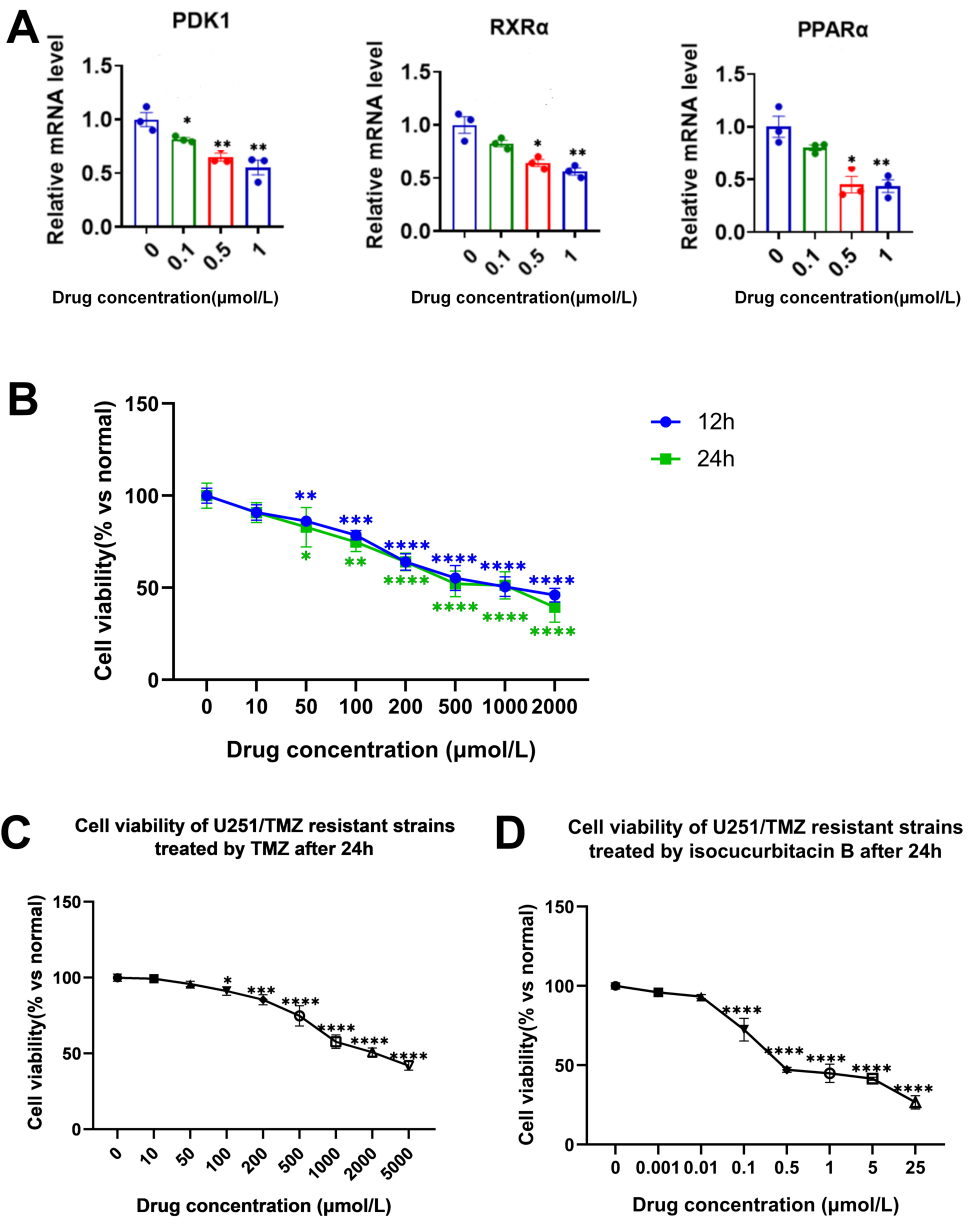
(A) Expression of pdk1, RXRα, PPAα, and Bcl-2 RNA was detected using RT-qPCR (^*^P < 0.05, ^**^P < 0.01 *vs.* control group); (B) The viability of U251 cells after 12 and 24 h of TMZ treatment (^*^P < 0.05, ^**^P < 0.01, ^***^P < 0.001, ^****^P < 0.0001 *vs.* control group); (C) The viability of U251/TMZ resistant strains after 24 h of TMZ treatment (^*^P < 0.05, ^***^P < 0.001, ^****^P < 0.0001 *vs.* control group); (D) The viability of U251/TMZ resistant strains after 24 h of isocuB treatment (^*^P < 0.05, ^***^P < 0.001, ^****^P < 0.0001 *vs.* control group). RT-qPCR: Reverse transcription-quantitative polymerase chain reaction; TMZ: temozolomide; isocuB: isocucurbitacin B.

We demonstrated that isocuB inhibited the glioma pathway based on WB and PCR results. IsocuB was found to inhibit glioma growth by suppressing PDK1, Bcl-2, PI3K-AKT, and MAPK signaling pathways, as well as the activation of MMP-2/9. Our results showed that the level of p-MAPK1/3 protein decreased significantly, while the level of total MAPK1/3 protein remained unchanged in the groups treated with different concentrations. In addition, the levels of total PI3K and t-AKT protein decreased significantly, while the levels of p-PI3K and p-AKT (S473) did not change significantly in the groups treated with different concentrations. The protein levels of PDK1 were significantly decreased [Figure 7A]. Furthermore, p-STAT3 protein levels were significantly reduced in the groups treated with different concentrations, but total STAT3 protein levels remained unchanged [Figure 7B]. IsocuB reduced the mRNA expression levels of PDK1, RXRα, PPARα, and Bcl-2 in U251 cells as determined by an RT-qPCR assay [Figure 8A]. These results suggested that isocuB played an anticancer role through the PI3K-AKT and MAPK pathways.

#### IsocuB inhibited U251/TMZ resistant strains

We investigated the inhibitory effects of 12-hour and 24-hour TMZ on U251 glioma cells. The IC_50_ values for 12-hour and 24-hour TMZ were 1,055 and 819.4 µmol/L, respectively. These values were significantly higher than the IC_50_ of isocuB at 12 and 24 h. Therefore, we could conclude that isocuB was more effective than TMZ [Figure 8B]. We established a resistant strain of U251/TMZ with an IC_50_ of 2,413 µmol/L after 24 h of TMZ administration [Figure 8C]. The RI grades range from 1 to 5, indicating low drug resistance, 5 to 15, indicating moderate resistance, and above 15, indicating high resistance. Thus, the RI is 2.95 and we have established a low drug resistance. Then, we verified the inhibitory effect of isocuB on U251/TMZ-resistant strains with an IC_50_ of 1.01µmol/L [Figure 8D].

#### IsocuB increased the pharmacological sensitivity to TMZ with the regulation of hsa-mir-1286a expression levels

**Figure 9:**
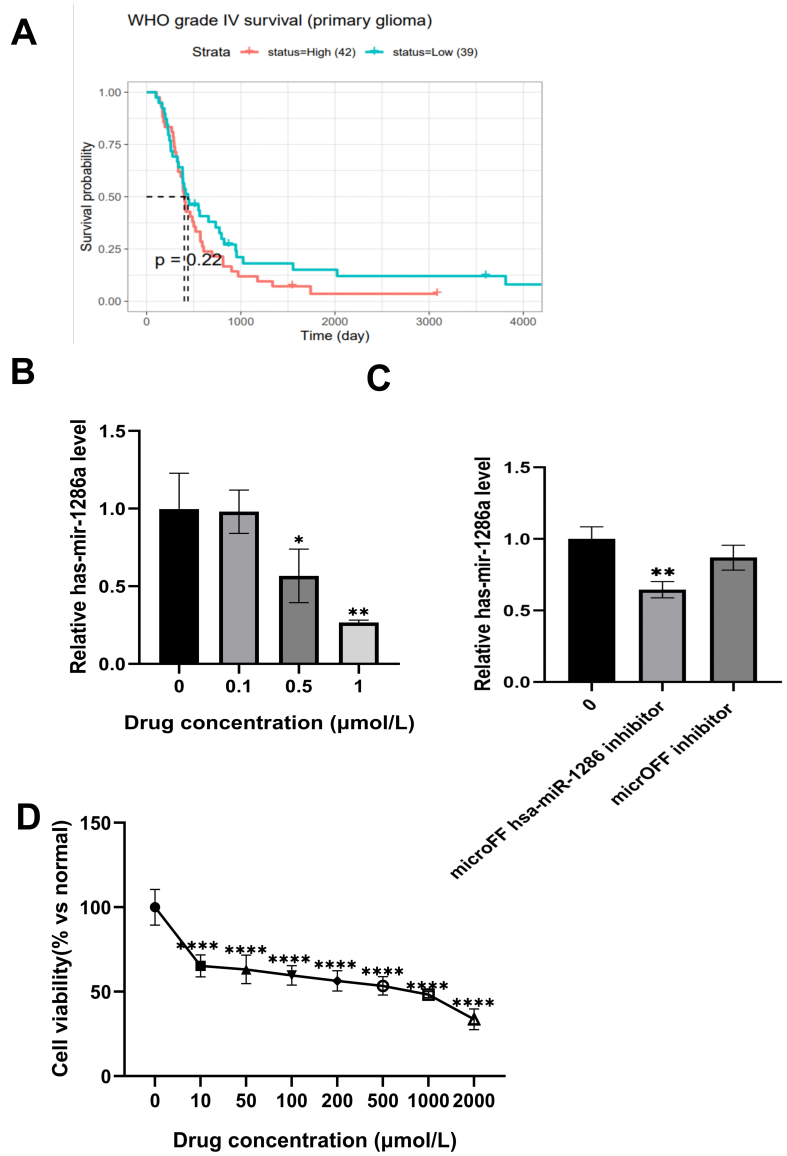
(A) Prognosis of low and high expression levels of hsa-mir-1286a in grade 4 gliomas; (B) Expression of hsa-mir-1286a was detected using RT-qPCR (^*^P < 0.05, ^**^P < 0.01 *vs.* control group); (C) Expression of hsa-mir-1286a after using hsa-miR-1286a inhibitor was detected using RT-qPCR (^**^P < 0.01 *vs.* control group); (D) Cell viability of U251 cells after 24 h of TMZ treatment after microFF hsa-miR-1286 inhibitor added (^****^P < 0.0001 *vs.* control group). RT-qPCR: Reverse transcription-quantitative polymerase chain reaction; TMZ: temozolomide.

Based on the literature, we hypothesized that hsa-mir-1286a would be associated with TMZ resistance after predicting the related miRNAs of AKT1 and MAPK1. Using the CGGM database, we examined the prognostic significance of hsa-mir-1286a in grade 4 gliomas and found that reduced expression was linked to better survival for these patients [Figure 9A]. The level of hsa-mir-1286a decreased steadily as the drug concentration increased according to the RT-qPCR data [Figure 9B]. Following the addition of micrOFF inhibitor NC and hsa-miR-1286a inhibitor, the RNA levels of hsa-mir-1286a decreased [Figure 9C]. Furthermore, the IC_50_ of U251 cells was significantly decreased by TMZ with the addition of the microRNA hsa-mir-1286a inhibitor; the IC_50_ was determined to be 441.2 µmol/L after 24 h [Figure 9D]. The RNA levels of hsa-mir-1286a decreased after the addition of micrON hsa-miR-1286a mimic [Supplementary Figure 1]. Moreover, after the addition of the micrON hsa-miR-1286a mimic, the IC_50_ of U251 cells was significantly reduced by TMZ, with the IC_50_ measured at 1,151 µmol/L after 24 h [Supplementary Figure 2].

#### IsocuB inhibited tumor growth in nude mice

**Figure 10:**
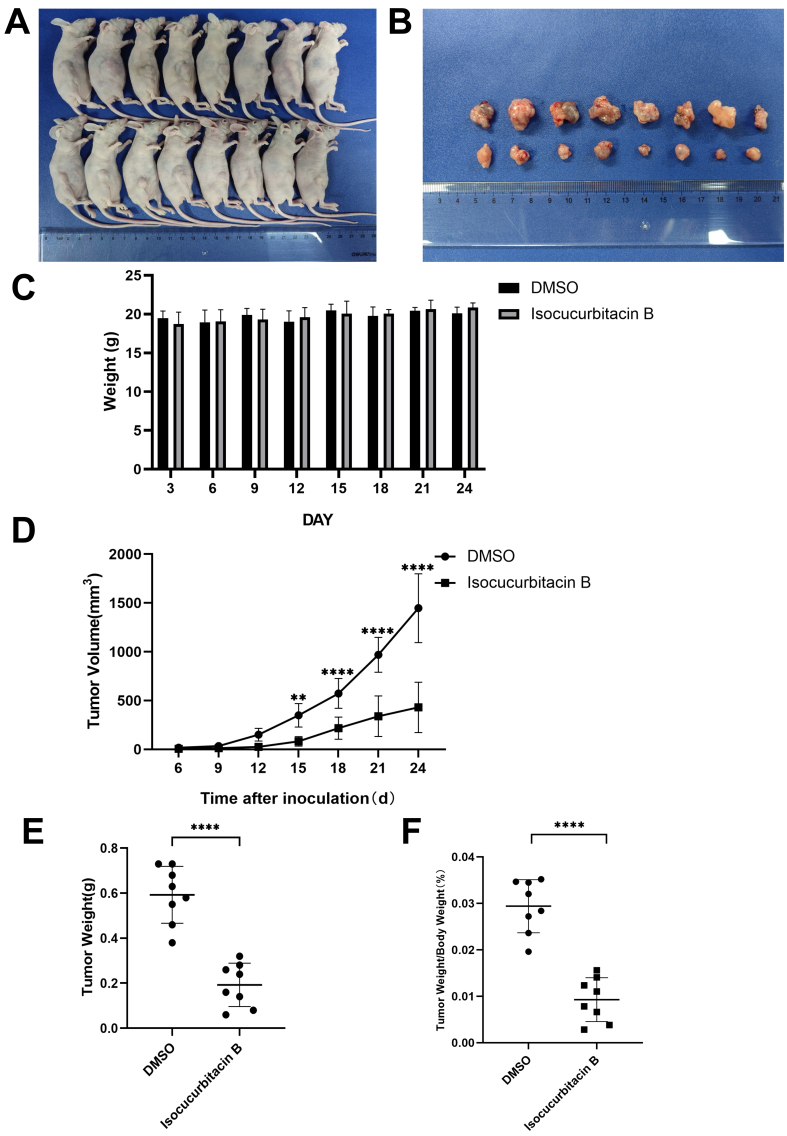
IsocuB inhibits tumor growth in nude mice. (A) Bossing of the mass in nude mice: the control group is shown above, and the treatment group is shown below; (B) Bossing: control group at the top and treatment group at the bottom; (C) Body weight changes in nude mice; (D) Daily tumor volume changes after administration (^**^P < 0.01, ^****^P < 0.0001 *vs.* control group); (E) The bossing weight (^****^P < 0.0001 *vs.* control group); (F) Bossing weight/body weight (^****^P < 0.0001 *vs.* control group). IsocuB: Isocucurbitacin B.

Previous studies of the toxicity of isocuB in C57BL/6 mice showed that the drug had no effect on the mice at doses below 2 mg/kg. Consequently, isocuB was administered to this experimental species at a dose of 2 mg/kg. After 18 days of intraperitoneal injection of 2 mg/kg/day of isocuB, the mass size was measured daily and the weight on the last day was recorded. “V (mm^3^) = L × W^2^/2” is the formula used to calculate the mass. Our results demonstrated that isocuB strongly suppressed cell proliferation. After administration of isocuB for 18 days (*P <* 0.0001), the weight of the mass decreased dramatically compared to the control group [Figure 10A-F]. Taken together, isocuB inhibited tumors *in vivo*.

## DISCUSSION

Accounting for 81% of all CNS malignancies, glioma is one of the most challenging malignancies to treat effectively. Recent studies have underscored the utility of exploring active compounds in natural medicinal materials as a promising strategy for developing novel medications^[7]^. IsocuB belongs to the class of terpenoids, which have been found to interact with a variety of signaling pathways, such as the MAPK, nuclear factor κB (NF-κB), and glycolytic enzyme pathways^[27]^. Namely, it is interesting to verify whether IsocuB may have anti-glioma properties.

Network pharmacology integrates systems biology, pharmacology, and computational analysis techniques to investigate the complex relationships between compounds, diseases, and targets^[28]^. Our study revealed 280 overlapping target genes of isocuB and glioma. Through PPI analysis, the top five genes were RXRα, AKT1, ESR1, MAPK1, and HSP90AA1. The GO and KEGG results revealed that the primary pathways targeted by isocuB against glioma were the PI3K-AKT and MAPK signaling pathways. AKT1 and MAPK1 were identified as the pivotal gene targets within these pathways, respectively. Previous studies have shown that the development, progression, and deterioration of various tumors are associated with the activation of the PI3K/Akt signaling pathway^[2,29]^. The MAPK signaling pathway, comprising several crucial signaling components and phosphorylation events, plays a pivotal role in tumorigenesis, including breast cancer^[30]^ and glioma^[31]^. Therefore, isocuB may exert its anti-glioma effect through the PI3K/AKT and MAPK signaling pathways. Molecular docking is a technique used in drug design to simulate receptor-drug interaction patterns. In recent years, the utilization of molecular docking to elucidate relevant mechanisms of action has become a trend in new drug development. Our results showed that isocuB exhibited a strong binding affinity for the top five genes, implying its potential in the growth of glioma through the action of these genes, which should be further analyzed and investigated. In this study, we analyzed the potential targets and related pathways of isocuB from a network pharmacology perspective.

Next, the genes and pathways through which isocuB acts were verified. A series of experiments confirmed that isocuB inhibited the proliferation, migration, and invasion of U251 and U87 cells, and induced apoptosis of U251 cells. Furthermore, the inhibitory effect of isocuB on U251 cells was significantly greater than that of TMZ. We hypothesized that it functions through the MMP family. The MMP family is associated with the survival, proliferation, apoptosis, invasion, and metastasis of cancer cells^[25]^. The WB and RT-qPCR results indicated a significant decrease in the mRNA and protein expression levels of MMP-2 and MMP-9. EMT is a process in which cells acquire invasive mesenchymal potential by undergoing a series of events such as loss of cell junctions, alteration of the cytoskeleton, and remodeling of the extracellular matrix^[26]^. The results showed a significant reduction in the protein levels of N-cadherin and Vimentin, indicating that isocuB could inhibit EMT and thus inhibit glioma invasion. Several studies have shown that the expression of MMP2/9 is regulated by the STAT3 signaling pathway in a variety of solid tumors, suggesting that increased activation of STAT is the cause of upregulation of MMP2/9 expression in cancer cells^[32,33]^. Our results further demonstrated that isocuB could impede glioma cell proliferation and migration by inhibiting STAT3 to inhibit MMP2/9. For specific pathways, WB and RT-qPCR experiments were performed for verification. Our study showed that isocuB could inhibit MAPK and STAT3 phosphorylation and PI3K-AKT non-phosphorylated expression, but not PI3K-AKT phosphorylated expression. Our results demonstrated that isocuB could inhibit U251 cells with the modulation of PI3K/AKT and MAPK signaling pathways. To further investigate the mechanism of action of isocuB, we verified the expression of pdk1 and Bcl-2 *in vitro* using RT-qPCR. The experiments *in vitro* showed that isocuB downregulated the protein and mRNA expressions by inhibiting PDK1, RXRα, PPARα, and Bcl-2. Different PPARs have significant effects on cancer^[34]^: PPARα is implicated in promoting proliferation, whereas PPARγ exhibits the opposite effect. Previous studies have linked PPARα to the promotion of the PI3K-AKT pathway^[35]^. Furthermore, *RXRα*^[36]^, *PDK1*^[37,38]^, and *Bcl-2*^[39,40]^ genes are involved in the PI3K/AKT and MAPK signaling pathways. These results further demonstrated that isocuB acts with the regulation of the PI3K/AKT and MAPK signaling pathways.

Hsa-mir-1268a is upregulated in both cells and exosomes of highly metastatic melanoma CSCs. In addition, the OL-ScS-derived exosome hsa-mir-1268a, after being taken up by OL cells, promoted the transfer and colonization ability of OL cells *in vitro* and *in vivo*. Therefore, inhibiting hsa-mir-1286a can inhibit tumor metastasis and colonization^[41]^. Then, through database search and literature review, we found that hsa-mir-1286a has great research potential in glioma resistance^[42]^, and hsa-mir-1268a was inhibited, and its inhibitors enhanced adriamycin-induced liver cancer cell death^[43]^. Our RT-qPCR experiment observed a gradual reduction in hsa-mir-1286a RNA content with increasing drug concentration. After inhibiting the expression of hsa-mir-1286a, the drug sensitivity of TMZ in U251 can be effectively improved. Then, we further established the U251/TMZ resistant strains, and the CCK-8 test found that isocuB could inhibit the growth of the resistant strains. *In vivo* experiments showed that isocuB inhibited tumor growth in nude mice. Our results collectively suggested that isocuB inhibited glioma cell proliferation, migration, and invasion, and induced apoptosis via the PI3K/AKT, MAPK, and STAT3 pathways. Our experiments showed that isocuB could reduce the level of hsa-mir-1286a in glioma U251 cells and increase the drug sensitivity to TMZ.

Although the above results exerted the inhibitory effect of isocuB on glioma, further study could be conducted to explore more detail about the anti-glioma effect and mechanism of isocuB. In the first place, the modulation of isocuB on hub genes in glioma is worthwhile to investigate further. We found the potential core targets of isocuB through network pharmacology and molecular docking, and verified the regulation of isocuB on hub genes via western blot and real-time PCR. However, the direct binding of isocuB to the core target has not been confirmed yet. Cellular thermal shift assay and MicroScale thermophoresis could be utilized for the confirmation of whether isocuB binds directly to the core target. Secondly, exploring the mechanism of isocuB in reversing drug resistance in glioma is necessary. We established the U251/TMZ-resistant strain, and found hsa-mir-1286a might play an important role in sensitizing drug-resistant cells to chemotherapy. Anyway, the combination effect of isocuB and TMZ, and the regulatory effect of hsa-mir-1286a on TMZ-resistant xenograft, as well as PI3K-AKT and MAPK pathways, are still unclear. In this situation, more experiments (e.g., calculating combined index) could be conducted with the combination of isocuB and TMZ in U251/TMZ-resistant strain. Adopting more resistant strains is beneficial for the study. For the animal study, the effect of isocuB on U251/TMZ-resistant model could be conducted in established TMZ-resistant xenograft. Transfection of hsa-mir-1286a mimic or inhibitor and addition of activator of PI3K-AKT and MAPK pathways benefit the investigation of the underlying mechanism of isocuB in TMZ-resistance. Thirdly, more animal models could be used in the study to give a more comprehensive evaluation of the effect of isocuB on glioma. We mainly utilized nude mice to establish the xenograft with a single dose administration. Actually, administration with different dosages in groups, at least dividing into high-dose group, medium-dose group, and low-dose group, could assess the dose-dependent effect of isocuB *in vivo*. Immunohistochemistry could be utilized to detect the protein expression *in vivo* and *in situ* hybridization could exhibit RNA expression, especially for hsa-mir-1286a, *in vivo*. Establishing a glioma-bearing mouse model via orthotopic transplantation of GL261 cells in C57BL/6 mice is also interesting for further study. Finally, according to our previous experiments, we found that isocuB was slightly toxic to mice. Ensuring the safety of innovative drugs is necessary for preclinical research. Thus, it is notable to conduct *in vitro* and *in vivo* experiments for evaluation and investigation of the toxicology of isocuB, and search for novel approaches for detoxification of isocuB, such as structural modification and addition of detoxicant.

Altogether, the underlying mechanism of anti-glioma activity of isocuB and detoxification of isocuB deserve further investigation, providing novel insights into the drug candidate in glioma treatment.

### Conclusions

The potential top five genes of the anti-glioma effect of isocuB were identified by network pharmacology as RXRα, AKT1, ESR1, MAPK1, and HSP90AA1. Molecular docking data further indicated that isocuB could bind to the corresponding protein of these genes with a good affinity. Moreover, AKT1 and MAPK1 mRNA were expressed at a high level in glioma, according to the clinical database analysis results. Furthermore, GO and KEGG analyses revealed that the PI3K/Akt and MAPK pathways were the top two connected pathways, as AKT1 and MAPK1 are the key genes of the PI3K/AKT and MAPK pathways. IsocuB exhibited superior efficacy over TMZ in inhibiting glioma proliferation, as evidenced by the CCK8 assay. IsocuB suppressed the invasion and migration of U251 cells by blocking the activity of MMP2 and MMP9 in wound healing and transwell assays. The TUNEL and FCM assays demonstrated the inhibitory effect of isocuB on BCL-2, thereby inducing cell death. Subsequently, we conducted RT-qPCR and WB assays to determine that isocuB could improve the sensitivity of U251 glioma to TMZ by inhibiting hsa-mir-1286a and reduce glioma growth with the modulation of PI3K/AKT and MAPK pathways. Ultimately, our findings illustrate that isocuB exerts an inhibitory effect on glioma growth and EMT process. The mechanism is associated with the regulation of PI3K/AKT and MAPK pathway. Additionally, it is interesting to find that isocuB enhances the drug sensitivity to TMZ via the decrease of hsa-mir-1286a RNA. The comprehensive evidence from experimental demonstration, database analysis, and network pharmacology supports the therapeutic potential of isocuB for glioma in the future.

## DECLARATIONS

### Availability of data and materials

The following supporting information can be downloaded.

### Copyright

© The Author(s) 2024.
