# Supplementary material for: Isocucurbitacin B inhibits glioma growth through PI3K/AKT pathways and increases glioma sensitivity to TMZ by inhibiting hsa-mir-1286a
Source: Cancer Drug Resist. 2024 May 8;7:16. doi: 10.20517/cdr.2024.01 (PMC11149100; doi:10.20517/cdr.2024.01)
Supplement: Supplementary file 1 [file cdr-7-16-SupplementaryMaterials.pdf]

Supplementary Materials

Isocucurbitacin B inhibits glioma growth through PI3K/AKT pathways and increases glioma sensitivity to TMZ by inhibiting hsa-mir-1286a

Mingyu Han[1], Junsha An[1], Sui Li[1,2,3,4], Huali Fan[1,5], Li Wang[1], Qing Du[6], Junrong Du[1], Yuxin Yang[2], Yuqin Song[3], Fu Peng[1]

[1]Department of Pharmacology, Key Laboratory of Drug-Targeting and Drug Delivery System of the Education Ministry, Sichuan Engineering Laboratory for Plant-Sourced Drug and Sichuan Research Center for Drug Precision Industrial Technology, West China School of Pharmacy, Sichuan University, Chengdu 610041, Sichuan, China.
[2]Department of Epidemiology and Health Statistics, West China School of Public Health and West China Fourth Hospital, Sichuan University, Chengdu 610041, Sichuan, China.
[3]Integrated Traditional Chinese and Western Medicine Department, Sichuan Clinical Research Center for Cancer, Sichuan Cancer Hospital & Institute, Sichuan Cancer Center, Affiliated Cancer Hospital of University of Electronic Science and Technology of China, Chengdu 610041, Sichuan, China.
[4]Waigaoqiao Free Trade Zone, WuXi Biologics, Shanghai 214122, China.
[5]Livzon Pharmaceutical Group Inc, Zhuhai 519090, Guangdong, China.
[6]Chongqing Western Biomedical Technology Co. Ltd., Chongqing, China.

Correspondence to: Yuxin Yang, Department of Epidemiology and Health Statistics, West China School of Public Health and West China Fourth Hospital, Sichuan University, Chengdu 610041, Sichuan, China. E-mail: 1581023993@qq.com; Yuqin Song, Integrated Traditional Chinese and Western Medicine Department, Sichuan Clinical Research Center for Cancer, Sichuan Cancer Hospital & Institute, Sichuan Cancer Center, Affiliated Cancer Hospital of University of Electronic Science and Technology of China, Chengdu 610041, Sichuan, China. E-mail: 774953409@qq.com; Dr. Fu Peng, Department of Pharmacology, Key Laboratory of Drug-Targeting and Drug Delivery System of the Education Ministry, Sichuan Engineering Laboratory for Plant-Sourced Drug and Sichuan Research Center for Drug Precision Industrial Technology, West China School of Pharmacy, Sichuan University, Chengdu 610041, Sichuan, China. E-mail: pengf@scu.edu.cn

Supplementary Table 1. Intersection target genes of isocucurbitacin B and glioma

**Gene**	ADH1C	MAPK10	DPP4	HEXB	SORD	CES1
MMP2	CTSK	CYP19A1	PCK1	WAS	NR1H4	IMPDH2
GP1BA	HNF4G	PPCDC	KDR	ANG	CFB	RHEB
MMP7	PDE4D	ITK	RORA	PSAP	MIF	GCK
TEK	CTSF	CA12	BACE1	DUSP6	AMD1	HCK
GRB2	STAT1	MMP3	RBP4	ADAM33	NOS2	F2
GSTP1	SDS	AMY1C	IL2	NQO1	MET	NR3C2
BST1	MAPK1	PPP5C	PAPSS1	RARG	ELANE	AMY1A
CMA1	CCNT1	SEC14L2	ACP3	FABP4	CD1A	MTAP
BLVRB	MAPK8	PNP	JAK3	HSD11B1	ADH5	REG1A
MDM2	HDAC8	KIF11	CA1	HSD17B1	SHBG	STS
AKT2	PDE4B	ABO	PAH	FGFR1	THRB	ADAM17
EGFR	FABP6	TNK2	MMP9	HSPA8	ALAD	YARS1
F10	PRKACA	CASP7	SOD2	CYP2C9	NQO2	SULT2B1
ABL1	CFD	CLK1	JAK2	BMP2	XIAP	EPHB4
PGR	UCK2	VDR	CASP1	ESR1	GSK3B	LTA4H
ALB	HPN	TTR	APOA2	CHIT1	PPARD	PDE3B
TREM1	DCK	PIK3CG	TYMS	MMP12	ACADM	PLAU
HINT1	EPHA2	SYK	MME	C1S	MMP8	FABP7
RNASE4	PLA2G2A	CDK5R1	CCNA2	ME2	PDE5A	PLK1
ERBB4	PNMT	CYP2C8	LGALS2	GLO1	CDK2	TNNC1
MMP13	GPI	AKT1	GSR	LGALS7	ITGAL	AKR1C2
MAPK14	PPARG	LCN2	CTNNA1	ARHGAP1	ATOX1	PPP1CC
TGM3	NR3C1	AR	PADI4	KYAT1	TGFB2	GM2A
HK1	CDK6	FABP5	TPSB2	DTYMK	RXRα	ARG1
CASP3	CSK	RARB	BCAT2	AKR1B1	RAC2	PPIA
PTPN1	LCK	GC	THRA	SRC	PKLR	PPARA
PYGL	FGFR2	HAGH	FECH	DPEP1	PITPNA	DHODH
ISG20	AMY2A	KIT	IMPA1	CCL5	FABP3	FKBP1A
NOS3	SULT2A1	ACE2	PIK3R1	AKR1C1	CSNK1G2	
SETD7	ANXA5	HMGCR	CSNK2A1	PRKCQ	NR1H2	
CBR1	EPHX2	HMOX1	ZAP70	LYZ	PDK2	
IGF1	PARP1	CRABP2	GSTA1	GSTM2	TGFBR1	
B3GAT1	RARA	PGF	GNPDA1	OAT	SHMT1	
HNMT	CA2	MAN1B1	CHEK1	PIM1	MAOB	
ALDH2	TTPA	FNTA	RNASE2	F11	CTSG	
ESRRA	FDPS	REN	SERPINA1	CTSS	RNASE3	
NR1I3	MAPKAPK2	TRAPPC3	AHCY	PDPK1	IGF1R	
MTHFD1	NMNAT1	CDA	ESRRG	CTSD	ST14	
CTSB	BCHE	C1R	SELE	HSP90AA1	MAP2K1	
FHIT	BIRC7	NR1H3	STAT3	ADK	AKR1C3	
BMP7	F7	PTPN11	TPH1	GSTA3	NR1I2	

Supplementary Table 2. Topological parameters of candidate targets

**Gene**	**Degree**	GSK3B	18	PPARG	8
SRC	84	PDPK1	18	GPI	8
PIK3R1	80	CSK	18	LCN2	8
MAPK1	74	AKR1C3	16	PCK1	8
AKT1	66	PIK3CG	16	ACE2	6
STAT3	66	AKT2	16	DPP4	6
GRB2	64	ZAP70	16	ADAM17	6
HSP90AA1	62	MMP2	16	ALDH2	6
PTPN11	60	ITK	16	PDE4B	6
RXRA	54	CDK6	14	CYP19A1	6
LCK	48	NOS3	14	MAOB	6
MAPK14	48	ALB	14	APOA2	6
EGFR	46	PRKCQ	14	ARG1	6
ESR1	44	EPHA2	14	CTSG	6
JAK2	40	MMP9	14	PARP1	6
MAPK8	36	PGR	14	KIF11	6
STAT1	34	FGFR2	14	CCNT1	6
CTNNA1	30	TGFBR1	14	PLK1	6
NR3C1	30	KIT	14	CDK5R1	6
PRKACA	30	CDK2	12	CTSD	6
HCK	28	NR1H3	12	EPHX2	6
IL2	28	TYMS	12	DUSP6	6
AR	28	TEK	12	HSPA8	6
JAK3	28	THRA	12	PLAU	6
ABL1	26	THRB	12	ELANE	6
MAP2K1	26	ADK	10	EPHB4	6
IGF1R	26	PDE3B	10	GP1BA	6
IGF1	26	AKR1B1	10	NR1H2	6
SYK	26	XIAP	10	HMGCR	6
MDM2	24	FABP6	10	HK1	6
RARA	24	DCK	10	PKLR	6
CASP3	24	VDR	10	PPP5C	6
PTPN1	22	PDE4D	8	SHMT1	6
PPARA	22	PNP	8	ACADM	4
ERBB4	22	RAC2	8	MME	4
NOS2	22	WAS	8	ADH5	4
F2	22	CASP7	8	GSTP1	4
KDR	22	HMOX1	8	AHCY	4
RARB	20	CHEK1	8	SORD	4
RARG	20	CDA	8	CBR1	4
MET	20	MMP3	8	AKR1C1	4
CCNA2	18	FGFR1	8	AKR1C2	4

SULT2B1	4	ANXA5	1	MAPK14	1
STS	4	OAT	1		
HSD17B1	4	BLVRB	1		
RHEB	4	NMNAT1	1		
FECH	4	CA2	1		
TTR	4	CCL5	1		
SERPINA1	4	UCK2	1		
SHBG	4	CRABP2	1		
BIRC7	4	CSNK1G2	1		
BMP2	4	CSNK2A1	1		
BMP7	4	CTSB	1		
FKBP1A	4	PSAP	1		
BST1	4	CYP2C8	1		
CASP1	4	CYP2C9	1		
FNTA	4	DTYMK	1		
CES1	4	MMP8	1		
RBP4	4	ESRRA	1		
MMP7	4	F7	1		
TNK2	4	FABP5	1		
F10	4	GC	1		
F11	4	GCK	1		
FABP4	4	PPP1CC	1		
PPARD	4	HDAC8	1		
NR1H4	4	NQO1	1		
FDPS	4	HNMT	1		
GNPDA1	4	NR3C2	1		
HSD11B1	4	ISG20	1		
PPIA	4	PAPSS1	1		
PGF	4	PNMT	1		
MAPKAPK2	4	ME2	1		
MAPK10	4	NR1I2	1		
MTHFD1	4	NR1I3	1		
PIM1	4	SDS	1		
RORA	4	TGFB2	1		
SULT2A1	4	SRC	1		
REN	2	PIK3R1	1		
ADH1C	2	MAPK1	1		
CCBL1	2	AKT1	1		
TPH1	2	STAT3	1		
PAH	2	GRB2	1		
PDK2	2	HSP90AA1	1		
SOD2	2	PTPN11	1		
ALAD	1	RXRA	1		
BCHE	1	LCK	1		

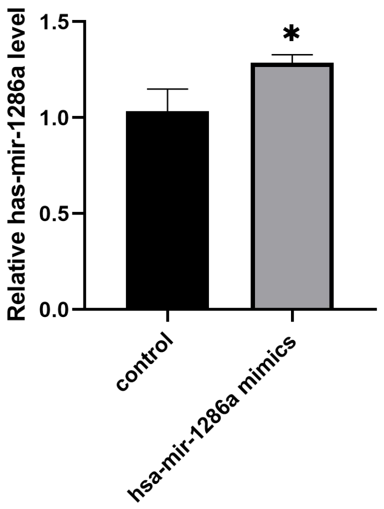

Supplementary Figure 1. Expression of hsa-mir-1286a after using micrON hsa-miR-1286a mimic was detected using RT-qPCR. ($^{**}P < 0.01$ *vs.* control group).

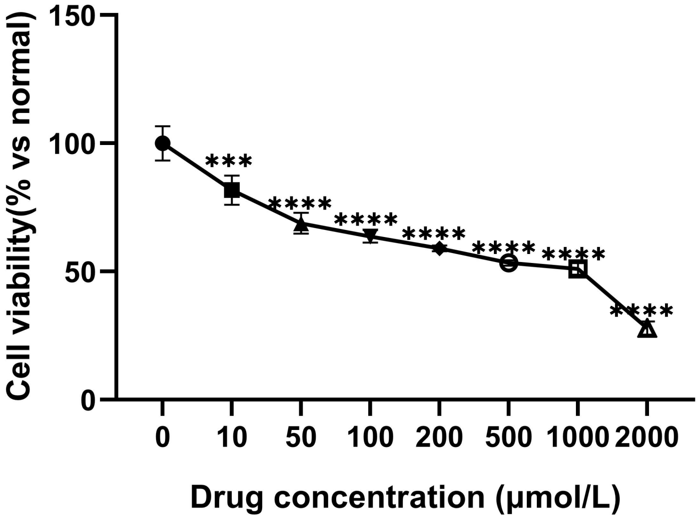

Supplementary Figure 2. Cell viability of U251 cells after 24 h of TMZ treatment after using micrON hsa-miR-1286a mimic. ($^{***}P < 0.001$, $^{****}P < 0.0001$ *vs.* control group).
